# Preterm Caesarean Delivery in a Parturient with *Candida parapsilosis* Endocarditis

**DOI:** 10.1155/2015/897645

**Published:** 2015-06-29

**Authors:** Jason Fu, Lance M. Retherford, Brigid Flynn

**Affiliations:** ^1^Department of Anesthesiology, Maimonides Medical Center, 4802 Tenth Avenue, Brooklyn, NY 11219, USA; ^2^Divisions of Cardiovascular Anesthesiology and Critical Care Medicine, The Permanente Medical Group, Inc., San Francisco, CA 94115, USA; ^3^Anesthesiology and Critical Care, Kansas University Medical Center, 3901 Rainbow Boulevard, Kansas City, KS 66160, USA

## Abstract

We present the first documented case of *Candida parapsilosis* infective endocarditis in a pregnant patient. While the incidence of infective endocarditis during pregnancy is rare, the incidence of *C. parapsilosis* endocarditis is even rarer. The numerous specific risks and decision making processes regarding this case are presented.

## 1. Case Presentation

A 23-year-old female, gravida 1, and para 0 at 31 weeks of gestation was transferred to our cardiothoracic intensive care unit (CTICU) for further management of her aortic valve endocarditis. Her past medical history included intravenous drug use (currently on methadone maintenance), hepatitis C discovered during this pregnancy, bipolar disorder, and depression with multiple prior suicide attempts. Of note, the patient also had a root canal two weeks prior and was started on ampicillin for a dental abscess.

**Figure 1:**
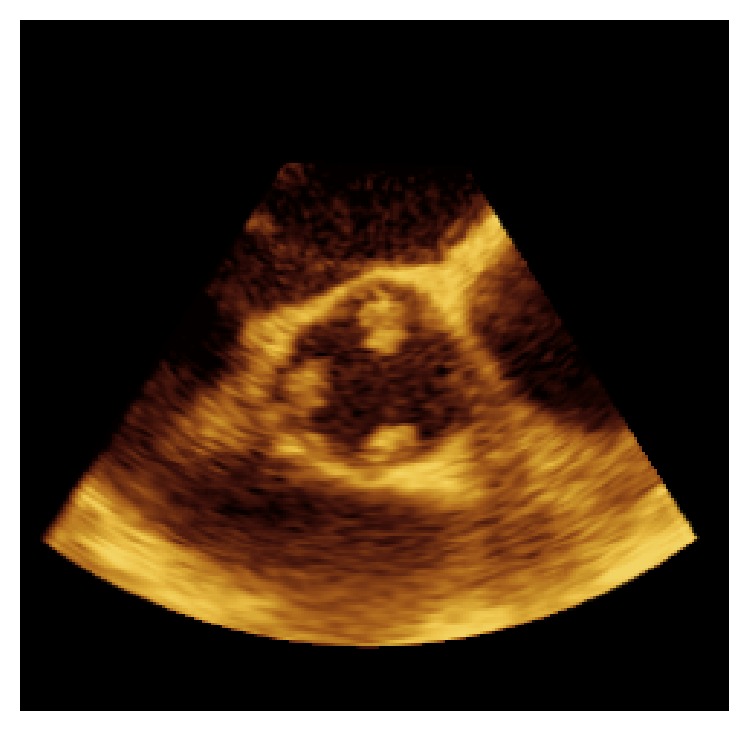
2D aortic valve short-axis, transesophageal view displaying fungal vegetation on all three aortic valve leaflets.

**Figure 2 fig2:**
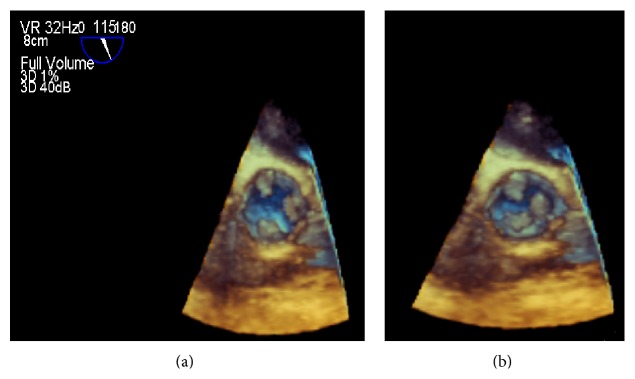
3D midesophageal aortic valve short-axis view. In (a), the valve is opening. Note the vegetation on all three leaflets. In (b), the valve is closed and vegetation can be seen obstructing the point of coaptation.

**Figure 3:**
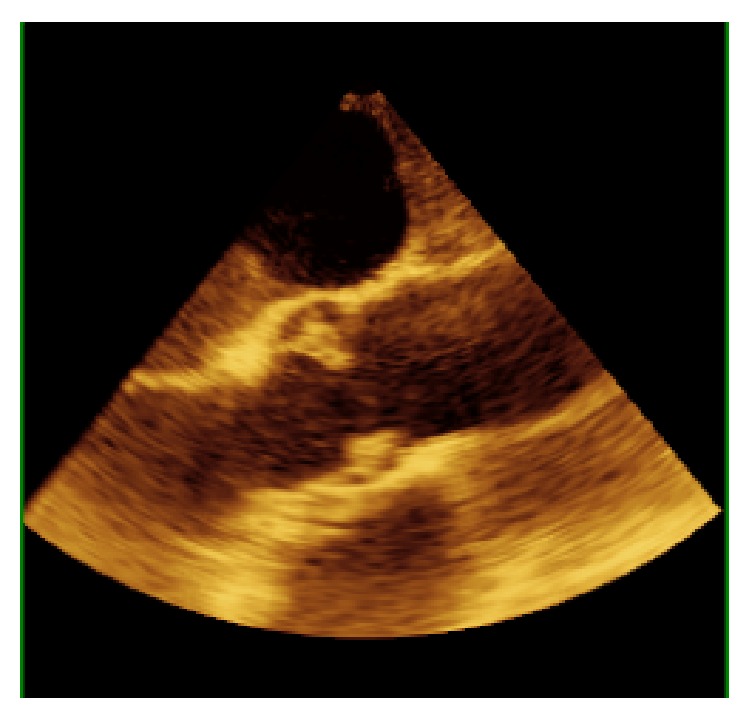
2D midesophageal aortic valve long axis with notable large vegetation.

She initially presented to an outside hospital one week earlier with severe right thigh and groin pain, which had progressed to the point of inhibiting ambulation. A right lower extremity Doppler study revealed an occlusive thrombus in the deep femoral artery. She was started on therapeutic enoxaparin and transferred to a second hospital, where a transthoracic echocardiogram (TTE) revealed large vegetative lesions on all 3 leaflets of the aortic valve, including a mobile vegetation measuring 0.8-1-cm on the ventricular side of the valve (Figures 1 [Fig fig2]–3). Mild to moderate aortic insufficiency was present. Blood cultures grew yeast but had not yet speciated upon transfer to our hospital. Amphotericin B was initiated and, after an initial infusion reaction, she was able to tolerate subsequent doses with premedication. Micafungin was added until speciation of the yeast was final. She remained hemodynamically stable requiring no inotropic or pressor support.

Anticoagulation was withheld despite her thrombus due to concern for cerebral embolic infarction and risk of hemorrhagic conversion in the setting of mobile aortic endocarditis. A magnetic resonance angiogram (MRA) of the brain was obtained to rule out a mycotic aneurysm, given that her endocarditis was known to be fungal in nature. The MRA showed a 2.2 mm aneurysm of the anterior communicating artery. An ophthalmologic exam was negative for fungal endophthalmitis. She received betamethasone and magnesium sulfate for fetal lung maturity and fetal neuroprotection, respectively. A fetal ultrasound demonstrated normal growth and activity of the fetus.

After a multidisciplinary meeting involving the CTICU intensivists, obstetrics, cardiothoracic surgery, cardiology, neurology, neurosurgery, obstetric anesthesiology, and obstetric and CTICU nursing teams, it was decided to proceed with delivery of the baby via Caesarean section followed by an aortic valve replacement (AVR) the following day. The decision to separate the procedures by approximately 12 hours was made out of concern for uterine bleeding after the Caesarean section with the full heparinization required for the AVR. It was decided that the risk of significant uterine bleeding, possibly necessitating a hysterectomy, would be too high in a 23-year-old woman.

The following day, the patient underwent a successful Caesarean delivery of a healthy baby under general endotracheal anesthesia. Her trachea was extubated so that she could spend time with her newborn. The following morning, she underwent a bioprosthetic AVR and a right femoral artery thrombectomy. Her blood cultures eventually speciated to* Candida parapsilosis*, as did intraoperative cultures.

It was decided that the patient would need long term antifungal therapy for her endocarditis and fungemia and that a peripherally inserted central catheter (PICC) was indicated. Initially there was a great deal of hesitation to place a PICC and possibly discharge her with one, given her history of intravenous drug abuse. Although there was no evidence of recent intravenous drug use, both on physical exam and on multiple urine toxicology screens, ethics and psychiatric consultations were obtained. After much deliberation, it was decided that PICC placement should not be withheld from her if it was medically indicated.

Discussions concerning whether the patient needed further brain imaging or intervention at some point for her aneurysm also took place. However, on further review of the MRA, the neurology service decided that based on its appearance and location the aneurysm was most likely congenital and not mycotic. Thus, no further imaging was warranted.

Postoperatively, the patient experienced considerable postsurgical pain that was difficult to control initially, likely due to her increased opiate requirements. In addition to her maintenance methadone dosing and a hydropmorphone PCA, she was maintained on fentanyl, ketamine, and dexmedetomidine infusions, which were gradually able to be weaned off over the next few days as her pain improved. The ketamine and dexmedetomidine infusions provided a multimodal and narcotic sparing approach to her opiate tolerant pain. She was eventually discharged from the CTICU on postoperative day 3 and from the hospital on postoperative day 17.

## 2. Discussion

As of 2007, there have been only 72 cases of* Candida parapsilosis* endocarditis documented [1]. Infective endocarditis during pregnancy is extremely rare as well, with an incidence of only 0.006% [2]. To the authors' knowledge, this is first case report of* Candida parapsilosis* endocarditis in a pregnant woman documented to date.

The maternal mortality rate with infective endocarditis during pregnancy can reach 33%, with most deaths related to heart failure or an embolic event (cerebral, coronary, or pulmonary). The rate of fetal mortality can reach 29%. In general, infectious endocarditis differs between intravenous drug abusers and nonusers in the fact that most intravenous drug abusers do not have underlying cardiac structural defects. Thus, the tricuspid valve is most commonly infected and the causative agent is usually* Staphylococcus aureus*. In patients with underlying structural cardiac defects, infectious endocarditis usually involves the left side of the heart and is caused by low-virulence bacteria, such as* Staphylococcus epidermidis*,* Streptococcus viridans*, and* Streptococcus faecalis* [3]. Although our patient had no underlying structural cardiac abnormality and was an intravenous drug abuser, she uncharacteristically developed fungal aortic valve endocarditis.

*Candida* is the most common cause of fungal endocarditis, with* Candida parapsilosis* being the most prevalent type of nonalbicans species isolated [4]. The most common predisposing factor for* Candida parapsilosis* endocarditis is the presence of prosthetic valves. The most common risk factor for infection of native valves is a history of intravenous drug use [1].* Candida parapsilosis* endocarditis carries high morbidity and mortality rates. Cerebral and peripheral embolic events occur in over 40% of patients, and overall mortality is over 40%. Lower mortality rates can be achieved with combined surgical and medical therapy, which underscores the importance of early diagnosis and multimodal treatment [1].

This case brings to light several issues concerning the parturient with cardiovascular disease. Much consideration went into the timing of the two necessary surgical procedures in this case. Consideration of performing only the AVR and allowing gestation to continue for several days to weeks would provide more time for pulmonary maturation. This technique has been used with success during the first reported case of maternal infective endocarditis [4]. In that case, an AVR was performed at 22 weeks of gestation and the baby was not delivered until term. However, subsequent reports were not as favorable. Montoya et al. documented a case of infective endocarditis in a patient who was 11 weeks of gestation and underwent an AVR. The patient fared well; however, the fetus died in utero [2]. In our case, we felt the risks imposed upon a fetus due to rapid hemodynamic changes and hypothermia during cardiopulmonary bypass would impose more significant risk than birth at 31 weeks of gestational age.

As far as performing the procedures concomitantly, the risk of uterine bleeding with the anticoagulation required for cardiopulmonary bypass is a concern. The treatment for massive uterine bleeding is ultimately a hysterectomy and in a young female, this risk has serious consequences. For this reason, it was decided that the AVR should occur after the Caesarean by several hours, allowing for evaluation of uterine bleeding via CTICU monitoring and an abdominal drain. Furthermore, waiting several hours allowed for assessment of mental status prior to the AVR in case of an embolic event. However, other authors have reported success with a mitral valve repair on the same day as a 33-week gestational Caesarean section in a patient with mitral valve endocarditis with no untoward events [5].

The obstetric anesthesia team involved decided the patient's fungemia and her recent systemic anticoagulation at the outside hospital for her deep femoral artery thrombosis were contraindications to neuraxial anesthesia. However, without any neuraxial anesthesia in place, there was concern that the potential hypertension and tachycardia associated with vaginal delivery would increase the risk of embolization of the vegetation with dire consequences. Additionally, it was felt that induction and trial of labor would only increase risk of cardiac dysfunction in the setting of aortic insufficiency. These risks were weighed heavily with the final decision electing for general endotracheal anesthesia as the safest measure. Her postoperative pain control was also an area of importance in this opiate tolerant patient and a multimodal analgesic approach consisting of her home methadone dosing, other opiates, and, importantly, ketamine and dexmedetomidine for the narcotic sparing properties of these agents [6].

## 3. Summary

This case describing a preterm parturient with* Candida parapsilosis* endocarditis illustrates that cardiac disease in the parturient can be managed safely. However, management requires a multidisciplinary team approach that must be individualized for each patient. In this case, we proceeded with Caesarean delivery of the fetus at 31-week gestational age, monitored the patient overnight, and then proceeded to an aortic valve replacement. While this patient did very well, each institution must weigh the numerous risks and benefits involving two patients (mother and baby) when making clinical decisions in cases of cardiac disease in the parturient.
